# Retrospective Analysis of Hatchling Success From Chelonian Eggs at a North Carolina Wildlife Clinic 2010 to 2023

**DOI:** 10.1002/zoo.70067

**Published:** 2026-04-15

**Authors:** Caroline C. Diehl, Sarah A. Hiser, Diane D. Moon, Christian M. Capobianco, Ronald K. Passingham, Ashley J. Kirby, Gregory A. Lewbart

**Affiliations:** ^1^ College of Veterinary Medicine North Carolina State University Raleigh North Carolina USA; ^2^ College of Veterinary Medicine University of Illinois Urbana Illinois USA; ^3^ University of Florida College of Veterinary Medicine Gainesville Florida USA; ^4^ Department of Clinical Sciences, College of Veterinary Medicine North Carolina State University Raleigh North Carolina USA; ^5^ Georgia Aquarium Atlanta Georgia USA

## Abstract

The success rate of deposited and harvested chelonian hatchlings between 2010 and 2023 were retrospectively reviewed by the North Carolina State University College of Veterinary Medicine Turtle Rescue Team. These data were analyzed to determine factors that may influence hatchling success including species, month of incubation, mother's status upon arrival, and the mother's presenting complaint. This study includes all wild chelonian species found in North Carolina that have presented as adult, gravid females over a 13‐year period. These species include common snapping turtles (*Chelydra serpentina*), painted turtles (*Chrysemys spp*.), yellow‐bellied sliders (*Trachemys scripta scripta*), cooters (*Pseudemys* spp.), eastern box turtles (*Terrapene carolina carolina*), eastern mud turtles (*Kinosternon subrubrum*), common musk turtles (*Sternotherus odoratus*), and red‐eared sliders (*Trachemys scripta elegans*). Overall, there were a total of 2453 chelonian eggs and of those, 954 (38.9%) hatched. The greatest egg count numbers were recorded from common snapping turtles (703/2453, 28.7%), eggs collected in May (1300/2453, 53.0%), eggs that were collected from patients euthanized on arrival (1084/2453, 44.2%), and eggs that came from mothers presenting for vehicular trauma (1891/2453, 77.1%). Trauma did not significantly impact overall hatch rates, except for snapping turtles with vehicle‐related injuries (*p* = 2.53e‐08). Eastern Box Turtles had significantly lower hatch ratios (*p* = 0.000157), while aquatic turtles, such as musk and yellow‐bellied sliders, fared better under current incubation protocols (*p* = 0.079 and *p* = 0.062, respectively). Results of this study represent opportunities to improve hatchling success and rearing under human care by understanding the major factors that contribute to their success.

## Graphical abstract

Retrospective analysis of chelonian eggs collected from gravid females admitted to a North Carolina wildlife clinic (2010–2023). A total of 2,453 eggs were harvested from live, euthanized, and deceased patients and incubated under standardized conditions, resulting in 38.9% hatching success. 55.5% of eggs that were collected and hatched came from mothers that were euthanized on arrival. Species was the only factor significantly associated with hatching success, while month of collection, maternal status, and presenting complaint were not significant. These findings demonstrate that egg harvesting and captive incubation are effective conservation strategies to support chelonian population recovery, particularly in cases involving anthropogenic trauma.

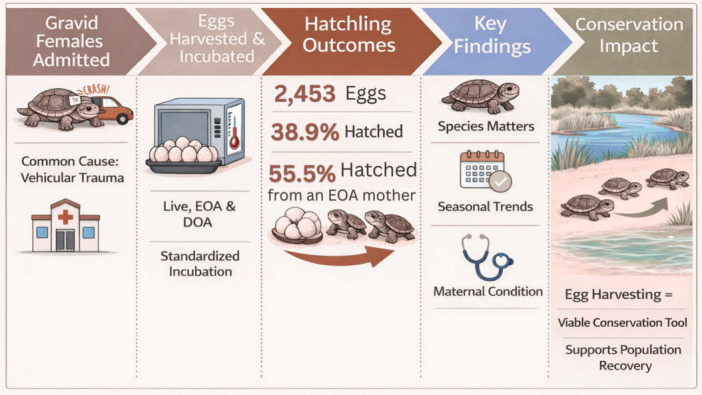

## 1 Introduction

In almost every species, the period following birth is when an individual is at the highest risk of mortality (Lawn et al. 2014). For chelonians, the most crucial survival period is from oviposition to hatching with significant risk of survival interference during this timeframe. Reptiles, in general, are characterized as having small distributional ranges and narrow niche survival requirements as compared with other vertebrates such as birds and mammals (Böhm et al. 2013). A downward population trend has been reported for reptiles over the last decade with 21% of reptiles considered threatened (Dambach et al. 2011; International Union for Conservation of Nature and Natural Resources IUCN 2023; Rhodin et al. 2018). Furthermore, an investigative report on the global conservation status of turtles and tortoise species specifically found that 20% of all recognized chelonian species are considered to be critically endangered and 56.3% of all species are at least threatened (Rhodin et al. 2018). Anthropogenic factors are major contributors to the negative population trends for chelonians (Erb et al. 2015). To combat these issues, there has been an increased effort in the captive rearing of endangered chelonians and other wildlife species, making the ability to improve captive rearing practices an important component of wildlife conservation efforts (Burghardt and Layne 1995; Tetzlaff et al. 2019).

The North Carolina State University College of Veterinary Medicine (NC State CVM) Turtle Rescue Team (TRT) is a veterinary student organization that has been treating sick and injured wild reptiles and amphibians since 1996, as well as prioritizes student education and clinical research (Lewbart et al. 2005). In addition to treating patients presented to the clinic, the TRT collects and incubates eggs that were laid or harvested from patients. Although incubating eggs is not uncommon in wildlife clinics that treat turtles, the success of this practice has not been investigated in any published studies to the authors' knowledge. Existing studies relating to turtle hatchlings primarily focus on experimental incubation conditions. Such conditions including environmental changes such as moisture and temperature have been found to affect hatching success and hatchling fitness in several reptile species (Bustard and Greenham 1968; Packard et al. 1987, 1989, 1991; McGehee 1990; Brooks et al. 1991; Janzen 1993; Bobyn and Brooks 1994; Spotila et al. 1994; Booth et al. 2004; Burgess et al. 2006). However, descriptive studies assessing the success of eggs incubated in captive clinical settings are lacking. This paper seeks to summarize all existing egg and hatchling data from past medical records at one wildlife medical facility and analyze the data to determine any factors that may affect successful hatching.

## 2 Methods

### 2.1 Data Collection and Study Design

This retrospective study analyzed chelonian egg collection and hatchling success data from cases at North Carolina State University College of Veterinary Medicine Turtle Rescue Team between 2010 and 2023. A case was defined as an adult, female, gravid, chelonian patient presenting to the clinic within this timeframe and this patient's medical record included the egg data. The medical records of these cases typically included species identification, patient status at the time of collection, maternal presenting complaint, number of eggs collected, hatch success. Species were reported by the intake manager and were identified to the lowest taxonomic level possible. In the event that species could not be identified, genus was provided and aggregated as a separate group. Patient status was defined as dead on arrival (DOA), euthanized on arrival (EOA), alive, or unknown in the case this was not documented. The presenting complaint was categorized according to the most severe injury upon arrival. This incorporated history, physical exam, and diagnostic workup. These were further classified into major categories: animal attack, aural abscess, eye pathology, fish hook, foreign body, hit by car (HBC), hit by lawnmower (HBL), head trauma, infection, limb trauma, shell trauma, none, other, or unknown in the event it was not documented. The number of eggs collected was recorded as a raw value in the medical record and reported as such. The hatch success was determined based on the reported number of eggs that hatched from that clutch and were documented in the medical record. Hatch successes were recorded as raw values in the medical record.

### 2.2 Statistical Analysis

Descriptive statistics were performed using Microsoft Excel 2023 (Microsoft, Redmond, Washington 98052, USA). For each factor (species, month, patient status, and presenting complaint), a summarized report was developed denoting the number of total cases, the number of eggs collected, and the number of eggs hatched for each year from 2010 to 2023.

Four statistical models were developed to assess factors influencing egg collection, hatch rates, and hatchling success:

#### 2.2.1 Model 1: Poisson Regression on Egg Collection

A Poisson regression model was used to examine the number of eggs collected as a function of species and presenting complaints. This model was chosen because the response variable (egg count) represents count data with a non‐negative integer distribution. The model equation is as follows: log(E(Y)) =\beta_0 +\sum_{i}\beta_{species_i} X_{species_i} +\sum_{j}\beta_{complaint_j} X_{complaint_j}

**Figure 1:**
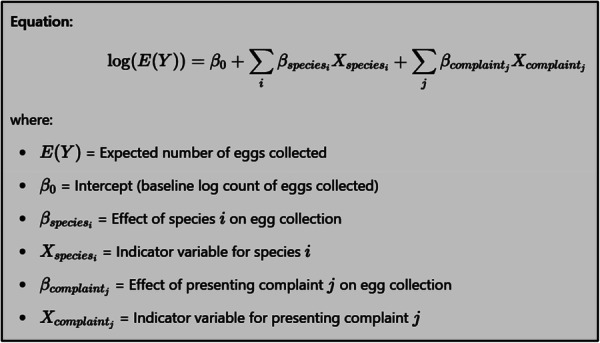
A poisson regression model used to demonstrate eggs collected as a function of species and presenting complaints.

**Figure 2:**
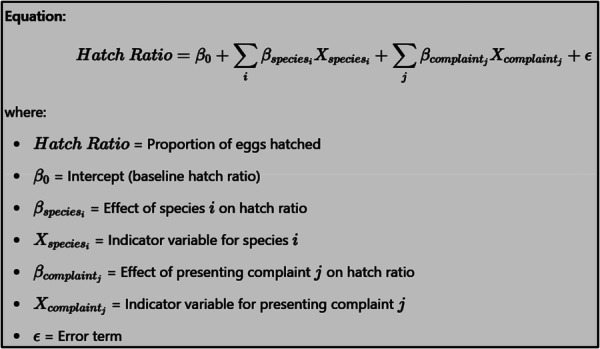
A generalized linear model with a Gaussian distribution was applied to examine hatch success ratios.

**Figure 3:**
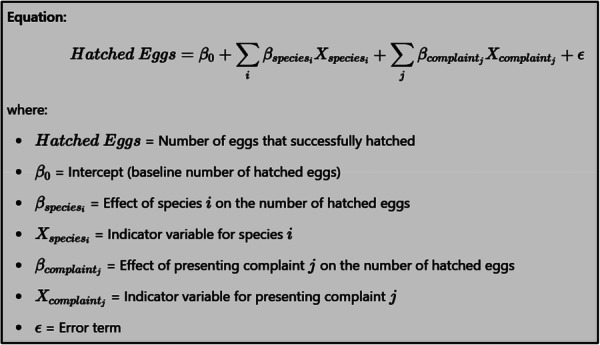
A linear regression model was employed to investigate factors affecting the absolute number of eggs that successfully hatched.

Significance testing was conducted using Wald chi‐square tests, and model fit was assessed using residual deviance and Akaike Information Criterion (AIC) (Figures 1, 2, 3).

#### 2.2.2 Model 2: Hatch Ratio Model

A generalized linear model (GLM) with a Gaussian distribution was applied to examine hatch success ratios. The model was specified as:

Hatch Ratio=β0 + ∑iβspeciesiXspeciesi + ∑jβcomplaintjXcomplaintj+ϵHatch\Ratio =\beta_0 +\sum_{i}\beta_{species_i} X_{species_i} +\sum_{j}\beta_{complaint_j} X_{complaint_j} +\epsilon

where ϵ\epsilon is the error term.

The primary outcome variable was the proportion of eggs that hatched within each species and maternal condition category. Model fit was evaluated using deviance reduction and AIC.

#### 2.2.3 Model 3: Number of Hatched Eggs Model

A linear regression model was employed to investigate factors affecting the absolute number of eggs that successfully hatched. The model followed the equation:

Hatched Eggs=β0 + ∑iβspeciesiXspeciesi + ∑jβcomplaintjXcomplaintj+ϵHatched\Eggs =\beta_0 +\sum_{i}\beta_{species_i} X_{species_i} +\sum_{j}\beta_{complaint_j} X_{complaint_j} +\epsilon

where variables are defined as in the previous models.

Model significance was assessed using *t*‐tests for individual predictors and overall model fit via residual sum of squares.

#### 2.2.4 Model 4: Interaction Effects on Hatched Eggs

A GLM with interaction terms was used to explore species‐specific responses to presenting complaints in hatch success. The model was structured as:

HatchedEggs=β0 + ∑iβspeciesiXspeciesi + ∑jβcomplaintjXcomplaintj + ∑kβinteractionk(Xspeciesk⋅Xcomplaintk)+ϵHatched\Eggs =\beta_0 +\sum_{i}\beta_{species_i} X_{species_i} +\sum_{j}\beta_{complaint_j} X_{complaint_j} +\sum_{k}\beta_{interaction_k} (X_{species_k}\cdot X_{complaint_k}) +\epsilon

**Figure 4:**
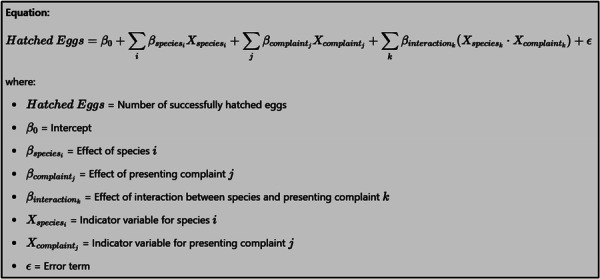
A generalized linear model with interaction terms was used to explore species‐specific responses to presenting complaints in hatch success.

**Figure 5:**
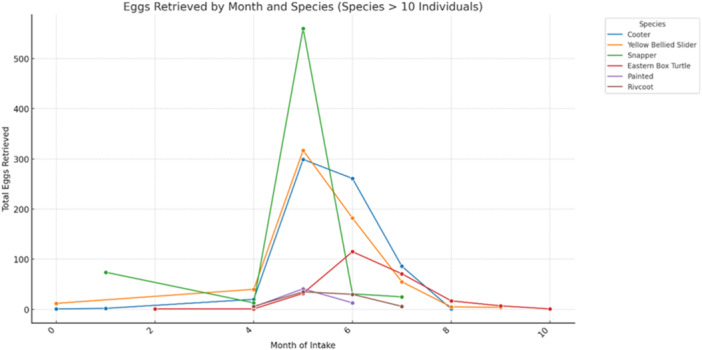
The species of chelonian eggs retrieved from adult, gravid female patients upon intake at the North Carolina State University College of Veterinary Medicine Turtle Rescue Team by month between 2010 and 2023.

**Figure 6:**
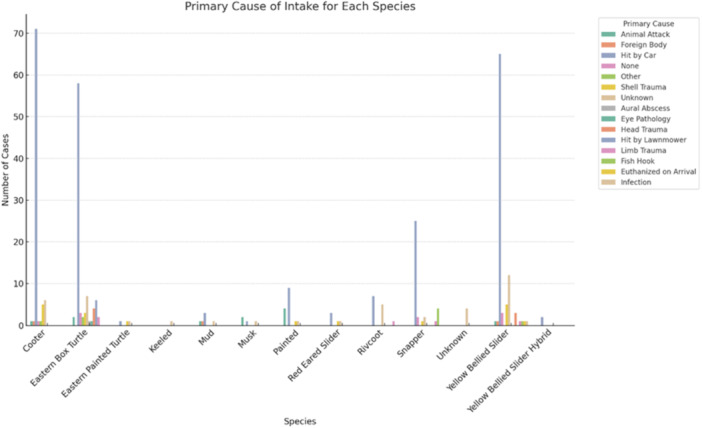
The presenting complaints in chelonian species representing adult, gravid, female patients upon intake at the North Carolina State University College of Veterinary Medicine Turtle Rescue Team between 2010 and 2023.

Interaction terms allowed for the evaluation of species‐dependent effects of maternal condition on hatching outcomes. Significance was assessed using likelihood ratio tests and AIC comparisons (Figures 4, 5, 6).

### 2.3 Software and Statistical Tools

All statistical analyses were performed using R (version 4.4.0) with the glm function for generalized linear models and the lm function for standard linear regressions. Model diagnostics included residual analysis, deviance comparisons, and collinearity assessments.

These methodological approaches provided robust statistical validation of factors influencing chelonian egg collection and hatch success, supporting evidence‐based conservation management strategies.

## 3 Results

### 3.1 Descriptive Statistics

Over the 13‐year study period (2010–2023), a total of 2453 collected chelonian eggs were documented in medical records. Of these, 954 (38.9%) hatched. There were 357 total cases reported of chelonian patients presenting to the clinic as an adult, female, and gravid. The greatest number of cases by species were yellow‐bellied sliders (94/357, 26.3%), eastern box turtles (87/354, 24.4%), and cooters (86/357, 24.1%). The months with the greatest number of presenting cases were May (129/357, 36.1%) and June (119/357, 33.3%). There were no reported cases in March, November, or December. The greatest number of these cases were live on arrival (177/357, 49.6%), followed by EOA (125/357, 35.0%), and DOA (24/357, 6.7%). Cases predominantly presented with an HBC injury (245/357, 68.6%).

The majority of eggs (2,445/2453, 99.7%) were attributed to known species. The species represented were common snapping turtles (*Chelydra serpentina*), painted turtles (*Chrysemys spp*.), yellow‐bellied sliders (*Trachemys scripta scripta*), cooters (*Pseudemys* spp.), eastern box turtles (*Terrapene carolina carolina*), eastern mud turtles (*Kinosternon subrubrum*), common musk turtles (*Sternotherus odoratus*), and red‐eared sliders (*Trachemys scripta elegans*). Common snapping turtles (*Chelydra serpentina*) contributed the highest number of eggs collected (703/2453, 28.7%). However, cooters (*Pseudemys spp*.) had the greatest number of successfully hatched eggs (332/954, 34.8%).

Eggs were collected from January to October. There were 2413 (98.4%) eggs that were associated with a known collection date in records. The greatest number of eggs were collected in May (1300/2453, 53.0%), and the number decreased each month progressing to October. There were no eggs collected in March, November, or December. May was also the month with the greatest number of eggs hatched (513/955, 53.8%).

There were 2284 (93.1%) eggs collected from chelonian patients with a documented status upon arrival. Eggs were collected from patients alive (deposited), euthanized, and dead (harvested). The greatest number of eggs were collected from patients who were EOA (1084/2453, 44.2%), followed by live patients (917/2453, 37.4%), and DOA (283/2453, 11.5%). Patients who were EOA also had the greatest number of eggs hatched (529/954, 55.5%).

A total of 2278 (92.9%) eggs were collected from chelonian patients with a documented presenting complaint. The presenting complaint that yielded the greatest number of eggs was vehicular trauma (1891/2453, 77.1%). There were 1935 (78.8%) eggs collected and incubated from incidents that involved known human interference (fish hook, HBC, or HBL). Patients who had a presenting complaint of an HBC had the greatest number of eggs that hatched (741/954, 77.7%) (Table 1).

**Table 1:** Chelonian species presenting as gravid, adult, female patients who had eggs naturally or collected at the North Carolina State University College of Veterinary Medicine Turtle Rescue Team by month between 2010 and 2023.

Species	Total individuals	Avg hatch ratio, %	Total eggs collected	Total eggs hatched	Avg eggs per individual
Cooter	86	42	670	332	7.9
Eastern Box Turtle	89	18	245	40	2.8
Eastern Painted Turtle	3	33	8	5	2.7
Keeled	1	0	4	0	4.0
Mud	6	50	8	4	1.3
Musk	4	75	5	4	1.3
Painted Turtles	15	23	59	17	3.9
Red Eared Slider	5	19	40	8	8.0
River Cooter	13	34	77	46	5.9
Snapper	35	32	703	187	20.1
Unknown	4	100	8	8	2.0
Yellow Bellied Slider	94	40	615	292	6.5
Yellow Bellied Slider Hybrid	2	100	11	11	5.5

### 3.2 Egg Collection Trends

The Poisson regression model demonstrated significant species‐specific differences in egg collection. Snapping turtles produced significantly more eggs than any other species (*p* < 2e–16), while eastern box turtles (*Terrapene carolina carolina*) had significantly fewer eggs (*p *< 2e–16). Presenting complaints also impacted egg collection, with gravid turtles experiencing vehicular trauma yielding the largest number of eggs (*n* = 1711). Among presenting complaints, trauma‐related conditions such as head trauma (*p* = 0.00035) and shell trauma (p < 5.67e‐06) were associated with higher egg counts.

### 3.3 Hatching Success by Species

The hatch ratio model revealed significant variability in hatching success across species. Eastern box turtles exhibited the lowest hatch ratios (*p* = 0.000157), while musk turtles and yellow‐bellied slider hybrids showed marginally higher hatch rates (*p* = 0.079 and *p* = 0.062, respectively).

### 3.4 Impact of Maternal Condition on Hatching

Analysis of hatched eggs showed that maternal injuries had minimal direct impact on hatching success. No significant difference in hatch rates was observed between eggs from live, euthanized, or dead‐on‐arrival (DOA) turtles. However, among injuries, eye pathology was associated with a significantly higher hatch ratio (*p* = 0.0169). Conversely, snapping turtles HBCs had significantly lower hatch success (*p* = 2.53e–08).

### 3.5 Influence of Month of Collection

**Figure 7:**
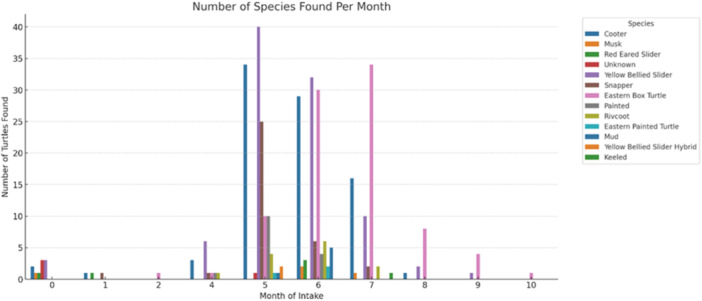
Chelonian species representing adult, gravid female patients upon intake at the North Carolina State University College of Veterinary Medicine Turtle Rescue Team by month between 2010 and 2023.

**Figure 8:**
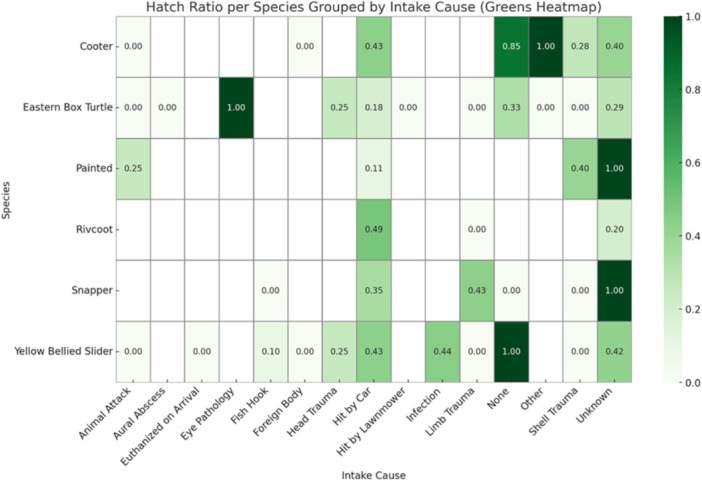
The hatch ratios per chelonian species representing adult, gravid female patients upon intake organized by presenting complaint at the North Carolina State University College of Veterinary Medicine Turtle Rescue Team between 2010 and 2023.

Egg collection occurred between April and October, with the highest number recorded in May (*n* = 1148), reflecting peak nesting seasonality. The highest hatch percentage occurred in September (57.1%) (Figures 7 and 8).

#### 3.5.1 Interaction Effects

The interaction model revealed that specific combinations of species and presenting complaints influenced hatching success. Snapping turtles without a recorded presenting complaint had significantly fewer hatched eggs (*p* = 1.03e–06), as did those with fish hook injuries (*p* = 0.0082).

**Table:** 

Key Findings and Implications
1. Species effects: Snapping turtles produced the most eggs, while cooters had the highest number of hatched eggs.
2. Injury effects: Trauma did not significantly impact overall hatch rates, except for snapping turtles with vehicle‐related injuries.
3. Seasonal trends: May had the highest number of eggs collected, while September had the highest hatch rate.
4. Hatching success factors: Eastern Box Turtles had significantly lower hatch ratios, while aquatic turtles fared better under current incubation protocols.
5. Conservation relevance: Results suggest that refining incubation strategies, particularly for species with lower hatch success, may improve outcomes.

## 4 Discussion

The analysis of hatchling success from chelonian eggs collected by the North Carolina State University College of Veterinary Medicine Turtle Rescue Team between 2010 and 2023 utilized multiple statistical models to determine factors influencing hatch success. These models included four generalized linear models for egg collection, hatch ratio, number of hatched eggs evaluation, and interaction effects between species and presenting complaints on egg hatch success. The findings from these models offer insights into species‐specific hatching success trends, the impact of maternal health conditions, and potential improvements in conservation strategies.

### 4.1 Egg Collection and Species Effects

The Poisson regression model analyzed factors influencing the number of eggs collected. The species with the highest number of eggs collected was the snapping turtle (*Chelydra serpentina*), which significantly outnumbered other species (*p* < 2e–16). Conversely, the Eastern Box Turtle (*Terrapene carolina carolina*) had significantly fewer eggs collected (*p* < 2e–16). The results align with natural reproductive tendencies. Snapping turtles typically have clutches of 20–73 eggs at a time, with a mean of 46.8 eggs (Iverson et al. 1997). Alternatively, eastern box turtles lay between 3 and 10 eggs in one clutch and yellow‐bellied sliders typically lay between 4 and 23 eggs at a time (Willey and Sievert 2012; Miller 2006).

### 4.2 Hatch Ratio and Species‐Specific Trends

The hatch ratio model revealed that species had a strong influence on hatching success. Eastern Box Turtles exhibited significantly lower hatch ratios (*p* = 0.000157), indicating reduced success prospects at the incubation stage. In contrast, musk turtles and yellow‐bellied slider hybrids showed marginally higher hatch ratios (*p* = 0.079 and *p* = 0.062, respectively). These findings suggest that environmental conditions in the incubator may be more conducive to species with naturally higher moisture or temperature tolerance. The current system maintains conditions most similar to those preferred by aquatic turtles such as cooters and sliders. Separate incubation chambers with modified parameters, including humidity, may be considered for each individual species. Understanding the species' differences provides the opportunity to improve hatchling success and captive rearing practices. In conjunction with experimental studies conducted, these studies demonstrate an opportunity to provide each chelonian species a more improved hatchling success rate.

### 4.3 Hatching Success and Presenting Complaints

The analysis of hatched eggs considered maternal presenting complaints to assess whether trauma or other medical conditions impacted reproductive success. Interestingly, no significant impact was found for injuries such as vehicular trauma (*p* = 0.0859), head trauma (*p* = 0.343), or shell trauma (*p* = 0.825), suggesting that eggs remain viable despite severe maternal injuries. However, eye pathology was associated with a significantly higher hatch ratio (*p* = 0.0169), which may indicate that less severe, non‐traumatic conditions have limited effects on embryonic development.

### 4.4 Influence of Month of Collection

Egg collection occurred between April and October, with the highest number recorded in May (*n* = 1148). This is consistent with the typical peak mating season in a natural setting (Palmer 1995). Turtle eggs have an average incubation period of 45–90 days, which was validated in this study (Nastiti and Ngurah 2013).

### 4.5 Interaction Effects and Influencing Factors

When analyzing interaction effects between species and presenting complaints, notable trends emerged. Snapping turtles HBCs had significantly lower hatch success (*p* = 2.53e–08), while those with no recorded presenting complaint also exhibited reduced hatching outcomes (*p* = 1.03e–06). Additionally, snapping turtles that had suffered fish hook injuries had markedly fewer hatched eggs (*p* = 0.0082). These results suggest that while trauma itself may not directly affect egg viability, species‐specific resilience to environmental stressors plays a role in reproductive outcomes.

Certain limitations exist in this study. From the period of 2010–2019, records were predominantly kept on paper. The period of 2020–2023 records is due to a switch to electronic records. Additionally, the ability to speciate an individual animal was dependent on the knowledge base of the case manager upon intake. To minimize errors, patients were aggregated to the genus level unless the species level was verified. In addition, there were minor differences in husbandry practices over the 13‐year period. Such differences include a new refrigerator and soil type discrepancies, and temperature regulation for limited species, such as switching between coconut fiber and natural North Carolina soil. Both soil types had successful and unsuccessful hatchling survivals but there could be minor effects due to this difference. The temperature range was set to 29°C. This is the proper range for cooters and yellow‐bellied sliders. While all other species have temperature specifications near this, it was not exactly their optimum temperature, which could have contributed to their chance of survival.

Future directions include continuing to investigate factors that impact hatchling success, such as treatment plans for the mother or various incubation standards for the hatchlings. Another factor to investigate is the length of time between death and egg harvest to determine how this affects viability. Oxytocin is sometimes used in the clinic to induce egg‐laying, and monitoring these cases may also provide additional information in future investigations. A similar analysis of data from other institutions, particularly those that see large volumes of reptiles, will demonstrate whether these findings are common across facilities. Examining fitness characteristics after hatch may also provide additional information about hatchling success since incubation conditions, particularly temperature and moisture, were found to affect other characteristics of hatchlings, such as sex, size, and locomotor performance (Packard et al. 1987; Packard et al. 1989; Packard et al. 1991; Janzen 1993; Spotila et al. 1994; Burgess et al. 2006). Temperature can be an especially important factor for species with temperature‐dependent sex determination, although the understanding of this process is still developing, and controlled conditions in the laboratory do not reflect the fluctuations found in nature (Carter et al. 2018). However, incubation temperature should ideally be regulated such that appropriately mixed sex ratios can result.

Chelonian populations have been declining, largely due to anthropogenic causes (Gibbons et al. 2000; Stanford et al. 2020). Turtles play an important ecological role and perform many ecosystem services; global declines in turtle populations can affect ecosystems in ways that are not fully understood currently (Lovich et al. 2018). Protecting populations of turtles is therefore not only important for individual species but for entire ecosystems. Although causes of population decline, such as habitat loss, need to be addressed, artificially collecting and hatching eggs can play a small role in propagating these populations. Overall, this research provides a strong foundation to future researchers to develop studies regarding species differences with regard to incubating eggs and hatching success. With the shift to captive rearing as a conservation initiative, this can lead to greater chances of survival for the hatchling and the species as a whole.

## Conflicts of Interest

The authors declare no conflicts of interest.
